# Identification and verification of feature biomarkers associated in heart failure by bioinformatics analysis

**DOI:** 10.1038/s41598-023-30666-0

**Published:** 2023-03-01

**Authors:** Yi-ding Yu, Yi-tao Xue, Yan Li

**Affiliations:** 1 Shandong University of Traditional Chinese Medicine, Jinan, 250014 China; 2 Affiliated Hospital of Shandong University of Traditional Chinese Medicine, Jinan, 250014 China

**Keywords:** Computational biology and bioinformatics, Computational models, Data mining

## Abstract

Heart failure is the final destination of most cardiovascular diseases, and its complex molecular mechanisms remain largely uncertain. This study aimed to systematically investigate the underlying molecular mechanisms and diagnostic and therapeutic targets of heart failure using bioinformatics. We obtained 8 healthy samples and 8 heart failure samples from GSE8331 and GSE76701. After removing the batch effect, we performed a differential analysis on it and obtained 185 differentially expressed ID. The results of enrichment analysis showed that the molecular mechanisms of heart failure were mostly related to immune, inflammation, and metabolism-related pathways. Immune cell infiltration analysis showed that the degree of infiltration of Tgd cells and Neurons was significantly enriched in heart failure samples, whereas pDCs and NKTs were in healthy tissue samples. We obtained Hub genes including EGR1, EGR2, FOS and FOSB by PPI network analysis. We established a 4-gene diagnostic model with Hub gene, and validated it in GSE21610 and GSE57338, and evaluated the discriminative ability of Hub gene by ROC curve. The 4-gene diagnostic model has an AUC value of 0.775 in GSE21610 and 0.877 in GSE57338. In conclusion, we explored the underlying molecular mechanisms of heart failure and the immune cell infiltration environment of failing myocardium by performing bioinformatic analysis of the GEO dataset. In addition, we identified EGR1, EGR2, FOS and FOSB as potential diagnostic biomarkers and therapeutic targets for heart failure. More importantly, a diagnostic model of heart failure based on these 4 genes was developed, which leads to a new understanding of the pathogenesis of heart failure and may be an interesting target for future in-depth research.

## Introduction

Cardiovascular disease is one of the leading causes of death in humans and includes high blood pressure, arrhythmia, coronary heart disease, heart failure and other heart-related diseases. Heart failure is the ultimate outcome of most cardiovascular diseases, mostly due to structural changes in the heart or functional disorders that impair ventricular filling or ejection function^1^. An estimated 64.3 million people worldwide have heart failure^2^. According to the observation data from Europe, the one-year all-cause mortality rate of heart failure exceeds 20%, which brings a heavy economic and social burden to the family and society^3^. Even with guideline-guided drug therapy, the prognosis for heart failure remains poor. Advanced patients often require frequent hospitalizations and lifelong medical management^4^. Therefore, early diagnosis of heart failure is of great significance for prognosis.

The pathological hallmark of heart failure is ventricular remodeling. As far as the information that can be collected from peripheral blood is concerned, although it has the advantages of simplicity and convenience in the early diagnosis of heart failure, it is difficult to detect structural changes in the heart. Imaging tests often do not detect ventricular remodeling until after it has occurred.Therefore, exploring the changes of myocardial gene expression in patients with heart failure is helpful for the early diagnosis of ventricular remodeling in heart failure, and the underlying molecular mechanism behind it also has the value of intervening in the treatment of heart failure. In addition, immune response also plays an important role in the development of heart failure. Infiltrating immune cells release cytokines such as TGF-β1 and TNF-α, which promote heart remodeling^5^. Therefore, exploring immune responses in the progression of heart failure could also help develop new diagnostic and therapeutic approaches.

With the rapid development of high-throughput technologies and bioinformatics, an increasing number of biomarkers show potential in the diagnosis and prognosis of heart failure, such as middle regional preatrial natriuretic peptide (MR-proANP), middle regional adrenomedullin (MR-proADM), highly sensitive troponin, soluble ST2 (sST2), growth differentiation factor (GDF)-15, Galectin-3, copeptin, Cystatin C (Cys-C) and Sirtuin (SIRT), however, more clinical evidence is needed before these markers can be used in clinic^6^. In addition, even for the same disease, different data sets and different analysis methods may produce different analysis results. For example, the Vijayakrishna Kolur team concluded that HF is related to the adaptive immune system and neutrophil degranulation by analyzing GSE141910, and obtained 10 characteristic genes^7^. Yang et al.^8^ analyzed GSE21610 and believed that HF is related to G protein-coupled receptor binding, peroxisome and cAMP signaling pathways, and obtained 5 characteristic genes. Therefore, our work can complement theirs to gain a more comprehensive understanding of the molecular mechanisms of heart failure.

In this study, we analyzed 2 datasets from GEO databases. Through systematic bioinformatics analysis, we obtained differentially expressed genes (DEGs) between heart failure myocardial samples and healthy heart samples, and explored the underlying pathological mechanisms of heart failure through functional enrichment analysis and PPI network analysis. We analyzed immune infiltration in failing myocardium to help us understand the role of immunity in the development of heart failure. We also constructed a four-gene diagnostic model by logistic regression analysis and validated the diagnostic model's validity in two other datasets.This study explores the underlying pathogenesis of heart failure and hopes to provide potential targets for the diagnosis and treatment of heart failure in the future.

## Materials and methods

### GEO datasets

The expression dataset of cardiac RNAs was collected from the online GEO database (www.ncbi.nlm.nih.gov/geo/). The initial search used the keywords "Heart failure", "Homo sapiens", and "expression profiling by array". We choose GSE8331 and GSE76701 datasets of GPL570 platform as model sets. The validation set selects the same platform datasets, GSE21610 (from GPL570 platform), and other platforms datasets, GSE57338^9–12^. GSE8331 contains 4 normal human heart samples and 4 failed heart samples with reduced ejection fraction. GSE76701 also contains 4 normal hearts and 4 failing hearts. GSE21610 contains 8 samples of normal hearts, 30 samples of failing hearts before ventricular assist devices (VAD) support and 30 samples of hearts after VAD support. We select 8 normal heart samples and 30 failing heart samples before VAD support as the validation set. GSE57338 is a dataset of 313 samples consisting of normal samples, ischemic left ventricle samples, and idiopathic dilated CMP left ventricle samples. We select 85 healthy samples and 72 failed heart samples among them as the validation set.

### Data preprocessing and DEGs identification

We used R software (R version 4.2.0) for data preprocessing. Download GSE8331 and GSE76701 from the GEO database via the GEOquery package. Remove probes that correspond to multiple molecules for one probe. For multiple probes corresponding to the same molecule, only the probe with the largest signal value is retained. The filtered data will use the ComBat function of the sva package to remove batch differences. Check the standardization through the box plot, and check the clustering between the sample groups through the PCA plot and the UMAP plot. We used the limma package for differential analysis of heart failure samples and normal samples, and selected genes with *p*-value < 0.05 and |log2(FC)|≥ 1 as differential genes. Use the ggplot2 package and the pheatmap package to complete the drawing of the picture^13–16^.

### Function and pathway enrichment analysis

To further analyze the function of myocardial expression profiles, we performed Gene Ontology (GO) and Kyoto Encyclopedia of Genes and Genes (KEGG) enrichment analysis of DEGs using the clusterProfiler package^17–20^. Use the GOplot package to combine the log2(FC) of the differential genes to calculate the Z-score value corresponding to each entry^21^. To avoid ignoring genes that are not significantly different but have important functions, we performed Gene Set Enrichment Analysis (GSEA) on all genes^22^.

### Immune cell infiltration analysis

We used the xCell package (It was validated using extensive in-silico simulations and cytometry immunophenotyping.) to assess the content of immune and stromal cells in cardiac samples to delineate the cellular heterogeneity landscape of cardiac expression profiles^23^. Differences in cell distribution between the heart failure group and the normal group were compared by t-test, and the cut-off value was set at *p* < 0.1. In addition, we used Spearman's correlation coefficient to evaluate the strength of correlation between cells. The visualization part is done using the ggplot2 package.

### Protein–protein interaction network and gene module identification

We uploaded the list of DEGs to the STRING database (string-db.org) to detect protein interactions with the following settings: Network type: full STRING network, meaning of network edges: evidence, minimum required interaction score: medium confidence (0.400)^24^. Subsequently, we imported the results into Cytoscape 3.6.1 for visualization and subsequent analysis^25^. In Cytoscape, we use the MCODE plugin to identify highly interconnected clusters with cutoff parameters set as follows: degree cutoff = 3, node score cutoff = 0. 2, k-Core = 2, Max. Depth = 100. The Hub gene was further screened with MCODE score > 4 as the standard^26^.

### Hub genes verification and diagnosis model construction

We validated the Hub genes screened in the PPI network in GSE21610 (30 heart failure samples and 8 healthy samples) and GSE57338 (85 healthy samples and 72 heart failure samples). We used the pROC package to build diagnostic models of single genes and combined genes, and evaluated the discriminative ability of key genes and diagnostic models by ROC curves.

## Results

### Data preprocessing and DEGs identification

**Figure 1:**
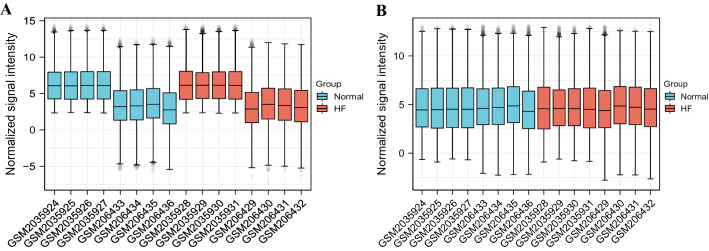
Boxplots of gene expressions before and after standardization for 2 selected GEO Database. ((**A**) Before standardization; (**B**) After standardization. Normal: Normal Group; HF: Heart Failure Group).

**Figure 2:**
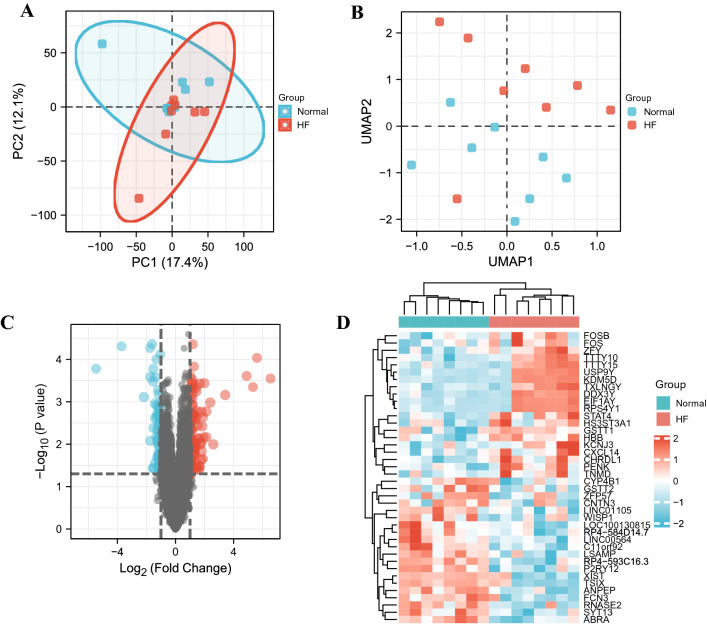
The PCA plot, UMAP plot, Volcano plot and Heatmap plot of gene expressions for 2 selected GEO Database with a screening criteria of |log2 FC|≥ 1 and adjust *p* value < 0.05. ((**A**) PCA plot; (**B**) UMAP plot; (**C**) Volcano plot; (**D**) Heatmap plot. PCA: Principal Component Analysis; UMAP: Uniform Manifold Approximation and Projection; Normal: Normal Group; HF: Heart Failure Group).

We combined the expression profiles of healthy heart samples and failing heart samples from GSE8331 and GSE76701 datasets of the GPL570 platform into a merged dataset containing 8 healthy samples and 8 disease samples. As shown in Fig. 1, we effectively remove batch effects between datasets by normalizing. After differential analysis, we obtained a total of 185 IDs with *p*-value < 0.05 and |log2(FC)|≥ 1, including 136 mRNAs, 46 LncRNAs and 3 unclassified genes. Figure 2 shows the PCA plot and UMAP plot of clustering among sample groups, as well as the volcano plot and heat map of differential IDs.

### Functional enrichment analysis

#### GO and KEGG enrichment analysis

**Figure 3:**
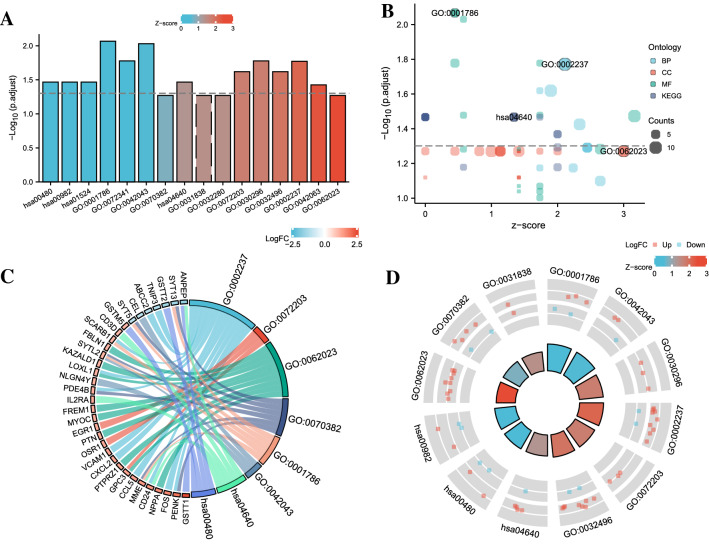
Enrichment plots by GO and KEGG. ((**A**) Bar graph, (**B**) Bubble plot, (**C**) chord diagram, (**D**) loop graph. BP: Biological Process; CC: Cellular Component; MF: Molecular Function).

To evaluate the function of DEGs, we combined the log2(FC) of DEGs to perform GO and KEGG enrichment analysis, and the results are shown in Table 1. Categories of GO analysis include biological process (BP), cellular component (CC), and molecular function (MF). Figure 3 shows the enrichment results of GO and KEGG. The mainly enriched BP terms included response to molecule of bacterial origin, cell proliferation involved in metanephros development, response to lipopolysaccharide, and gliogenesis. The mainly enriched CC terms contained collagen-containing extracellular matrix, exocytic vesicle, haptoglobin-hemoglobin complex, and symmetric synapse. The mainly enriched MF terms were phosphatidylserine binding, neurexin family protein binding, protein tyrosine kinase activator activity, and modified amino acid binding. In KEGG pathway enrichment analysis, DEGs mainly enriched in Hematopoietic cell lineage, Glutathione metabolism, Drug metabolism—cytochrome P450, and Platinum drug resistance.

**Table 1:** Significant enriched GO terms and KEGG pathways of DEGs.

Ontology	ID	Description	GeneRatio	BgRatio	*p* value	p. adjust	q value
BP	GO:0,002,237	Response to molecule of bacterial origin	11/114	343/18,670	8.17e-06	0.017	0.015
BP	GO:0,072,203	Cell proliferation involved in metanephros development	3/114	10/18,670	2.58e-05	0.024	0.021
BP	GO:0,032,496	Response to lipopolysaccharide	10/114	330/18,670	3.48e-05	0.024	0.021
BP	GO:0,042,063	Gliogenesis	9/114	290/18,670	7.252E-05	0.038	0.033
CC	GO:0,062,023	Collagen-containing extracellular matrix	9/116	406/19,717	6.68e-04	0.054	0.050
CC	GO:0,070,382	Exocytic vesicle	6/116	207/19,717	0.001	0.054	0.050
CC	GO:0,031,838	Haptoglobin-hemoglobin complex	2/116	11/19,717	0.002	0.054	0.050
CC	GO:0,032,280	Symmetric synapse	2/116	11/19,717	0.002	0.054	0.050
MF	GO:0,001,786	Phosphatidylserine binding	5/109	58/17,697	2.86e-05	0.009	0.008
MF	GO:0,042,043	Neurexin family protein binding	3/109	13/17,697	6.21e-05	0.009	0.008
MF	GO:0,030,296	protein tyrosine kinase activator activity	3/109	19/17,697	2.05e-04	0.017	0.015
MF	GO:0,072,341	Modified amino acid binding	5/109	89/17,697	0.0002	0.017	0.015
KEGG	hsa04640	Hematopoietic cell lineage	5/52	99/8076	4.12e-04	0.034	0.030
KEGG	hsa00480	Glutathione metabolism	4/52	57/8076	4.69e-04	0.034	0.030
KEGG	hsa00982	Drug metabolism—cytochrome P450	4/52	71/8076	0.001	0.034	0.030
KEGG	hsa01524	Platinum drug resistance	4/52	73/8076	0.0012	0.034	0.030

#### GSEA enrichment analysis

**Figure 4:**
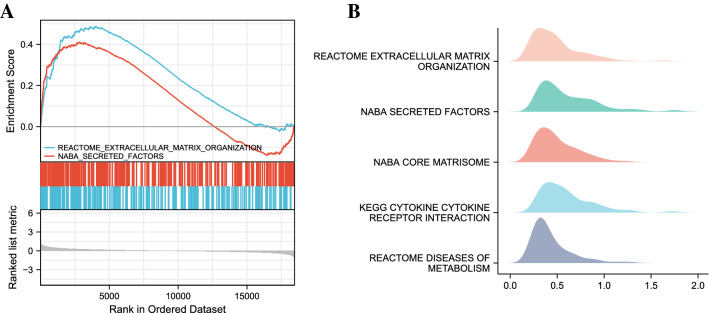
Enrichment plots by GSEA. (**A**) GSEA Visual Analysis, (**B**) GSEA ridgeplot. NES: Normal Enrichment Score; FDR: False Discovery Rate).

To assess the contribution of genes in the dataset in phenotypes, we performed GSEA enrichment analysis on them. The data set is C2.Cp, the random number seed is 2020, and the calculation is performed 1000 times. Each gene set contains between 10 and 500 genes. We visualized the top five datasets that met the criteria of FDR < 0.25 and *p*-value < 0.05, including Extracellular matrix organization, Genes encoding secreted soluble factors, Ensemble of genes encoding core extracellular matrix including ECM glycoproteins, collagens and proteoglycans, Cytokine-cytokine receptor interaction, and Diseases of metabolism. The visualization results are shown in Fig. 4, and the specific GSEA enrichment analysis information is shown in Table 2.

**Table 2:** GSEA enrichment analysis.

ID	Description	setSize	enrichmentScore	NES	*p* value	q values	rank
REACTOME_EXTRACELLULAR_MATRIX_ORGANIZATION	Extracellular matrix organization	291	0.49	2.09	0.001	0.17	3998
NABA_SECRETED_FACTORS	Genes encoding secreted soluble factors	313	0.41	1.77	0.001	0.17	2839
NABA_CORE_MATRISOME	Ensemble of genes encoding core extracellular matrix including ECM glycoproteins, collagens and proteoglycans	256	0.51	2.18	0.001	0.17	3565
KEGG_CYTOKINE_CYTOKINE_RECEPTOR_INTERACTION	Cytokine-cytokine receptor interaction	250	0.41	1.75	0.001	0.17	2800
REACTOME_DISEASES_OF_METABOLISM	Diseases of metabolism	227	0.42	1.75	0.001	0.17	3684

#### Immune cell infiltration analysis

**Figure 5:**
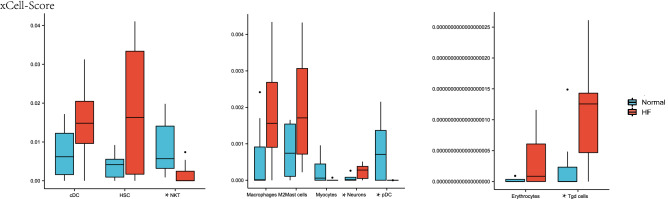
xCell scores of immune and stromal cells between HF and healthy heart tissues in merged dataset (*p < 0.05; Normal: Normal Group; HF: Heart Failure Group).

**Figure 6:**
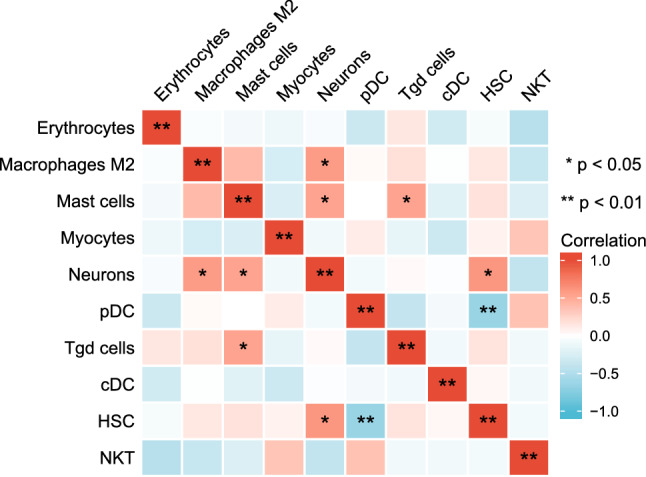
Correlation matrix of immune cells. Blue squares represent negative correlation, and red squares represent positive correlation.

xCell was used to assess cellular composition heterogeneity between heart failure samples and healthy samples. As shown in Fig. 5, 10 cell types were altered in heart failure heart tissue compared to healthy hearts, with 4 cell types significantly altered. Scores of cDC, HSC, Macrophages M2, Mast cells, Neurons, Erythrocytes, and Tgd cells were increased in heart failure heart tissue, whereas NKT, Myocytes, and pDC scores were decreased. In addition, we used Spearman's correlation coefficient to evaluate the strength of correlation between cells. As shown in Fig. 6, HSCs had the highest negative correlation with pDCs. Neurons had a high positive correlation with Macrophages M2, Mast cells, and HSC. Tgd cells had a high positive correlation with Mast cells.

#### PPI network construction and hub gene selection

**Figure 7:**
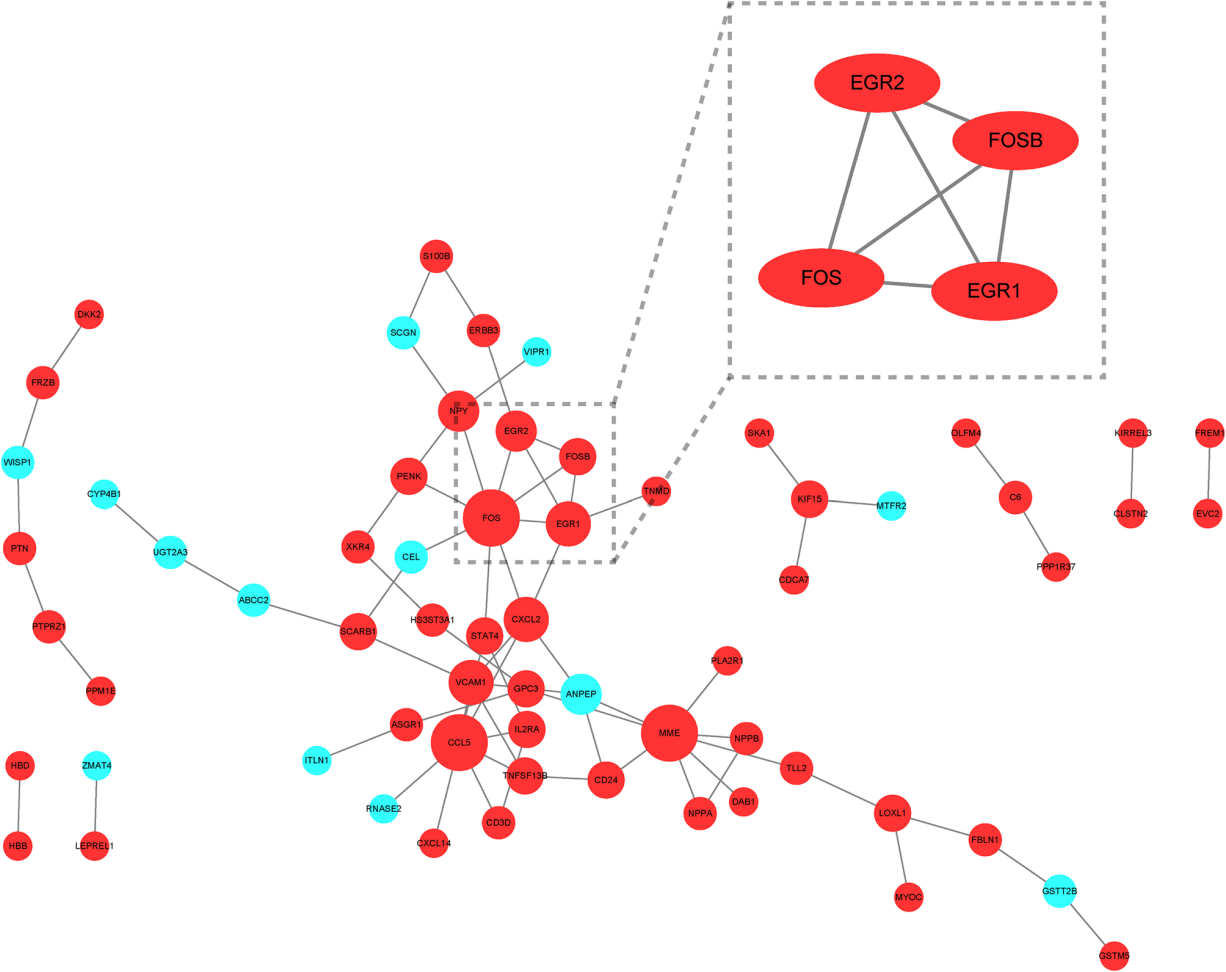
PPI network construction. (Red represents up-regulated genes, blue represents down-regulated genes).

We entered the DEGs into the STRING database for PPI network construction, and finally identified 64 nodes and 73 interactions. In addition, we identified a Hub gene module with 4 nodes and 6 edges by MCODE, and the results are shown in Fig. 7.

#### Hub genes verification and diagnosis model construction

**Figure 8:**
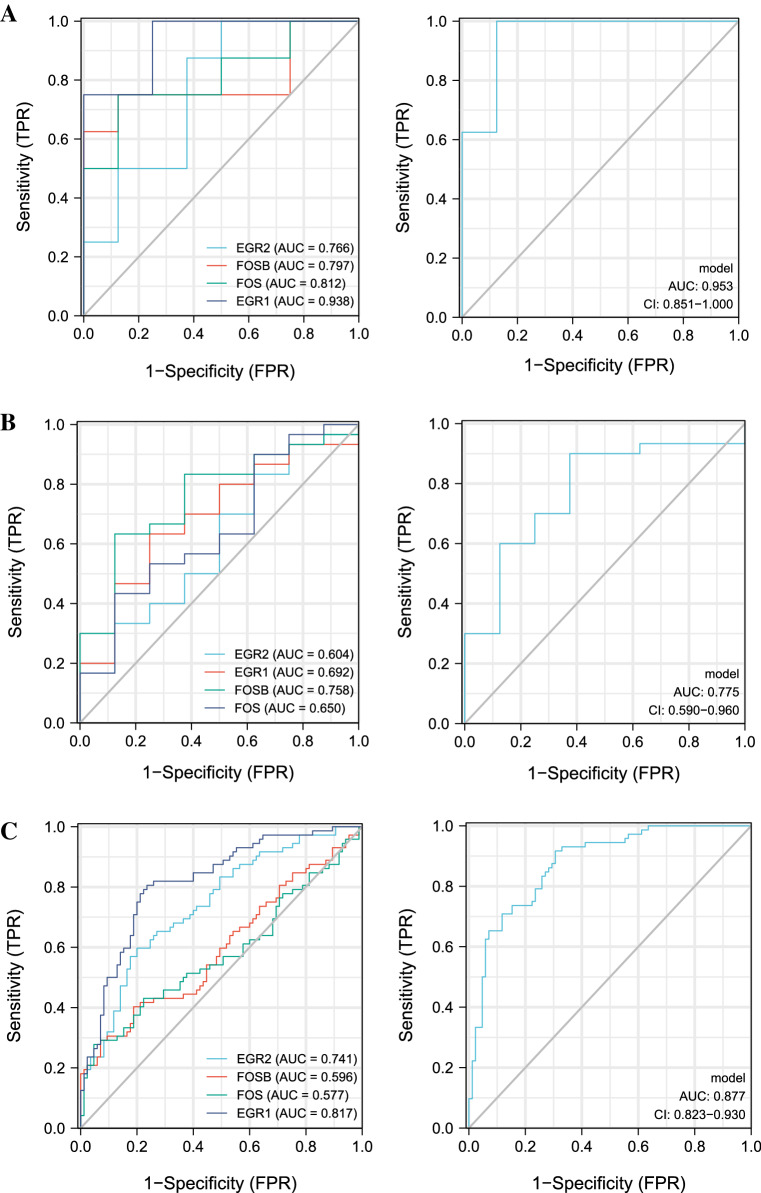
Analysis of the disease predictive abilities of the four selected hub genes. (**A**) ROC curve analysis of diagnostic models for single and combined genes in merged dataset. (**B**) ROC curve analysis of diagnostic models for single and combined genes in GSE21610. (**C**) ROC curve analysis of diagnostic models for single and combined genes in GSE57338.

We identified the composition of the Hub gene module as EGR1, EGR2, FOS and FOSB in the PPI network, all of which were up-regulated genes. We established ROC curve analysis to validate the diagnostic value of selected biomarkers. In the diagnostic model of a single gene, only EGR1 has high diagnostic value, and the AUC of other genes is not high. However, the combined genome has a high diagnostic value for heart failure. We constructed diagnostic models of Hub genes in different datasets by logistic regression and visualized them with ROC curves. The results are shown in Fig. 8.

## Discussion

HF is a major public health challenge. The use of drugs such as ARNI and dapagliflozin has also greatly improved the prognosis of heart failure. However, if the occurrence of heart failure can be detected earlier and the treatment can be intervened as soon as possible, the benefit of patients will be greater. In recent years, bioinformatics research has made great progress in the field of heart failure, and breakthroughs have been made in both the diagnosis of heart failure and the underlying molecular mechanisms. Integrating more biomarkers can comprehensively intervene in the diagnosis and treatment of heart failure. In addition, exploring related processes such as immune cells and pathways in the physiological and pathological processes of heart failure can also provide potential diagnostic and therapeutic targets to a certain extent.

In this study, we integrated 16 samples from 2 datasets and analyzed the data using bioinformatics.

In the GO enrichment analysis results, the main ones involved in heart failure were response to lipopolysaccharide, phosphatidylserine binding, and protein tyrosine kinase activator activity.

The cytokine hypothesis states that the progression of heart failure is attributable to sustained proinflammatory cytokine signaling. TNFα has been shown to induce cardiomyocyte dysfunction, cardiomyocyte fibrosis, and negative inotropic effects, and its elevation is associated with cardiac fibrosis, ventricular dilatation, and mortality^27,28^. Lipopolysaccharide is a cell wall component of Gram-negative bacteria and has a strong pro-inflammatory effect. Some researchers concluded that even a small concentration of lipopolysaccharide can effectively induce the release of TNFα in an in vitro whole blood model of heart failure patients^29^. Their study also demonstrated that response to lipopolysaccharide predicts survival in patients with chronic heart failure^30^. This suggests that maintaining the function of immune cells and the immune system is essential in the treatment of heart failure.

Phosphatidylserine is located on the cytoplasmic lobe of the plasma membrane bilayer. Energy loss during cellular injury or apoptosis results in disruption of plasma membrane asymmetry, resulting in surface exposure of phosphatidylserine. Subsequently, macrophages recognize damaged cells and complete the elimination of apoptotic cells. Modulation of membrane fluidity or phosphatidylserine binding can affect this process^31^. Cardiomyocyte apoptosis is a key regulator in the development of heart failure^32^. Therefore, phosphatidylserine can be employed as an early diagnostic indicator of heart failure. It may be also given potential therapeutic value in heart failure by modulating phosphatidylserine binding.

Tyrosine kinases are enzymes that catalyze the transfer of phosphate from ATP to tyrosine residues in polypeptides. The small molecule tyrosine kinase inhibitor imatinib is a targeted drug for the treatment of chronic myeloid leukemia, but its cardiotoxicity can cause heart failure. In cardiomyocytes, ABL functions to maintain endoplasmic reticulum homeostasis. Imatinib induces endoplasmic reticulum stress, activates the PERK signaling pathway, and produces mitochondrial dysfunction^33^. Therefore, maintaining the activity of protein tyrosine kinase activators may reduce the cardiotoxicity of antitumor drugs.

In the KEGG pathway enrichment analysis results, Glutathione metabolism and Drug metabolism—cytochrome P450 are mainly involved in heart failure. Abnormal intracellular glutathione metabolism is related to increased free radical production in chronic heart failure. Therefore, glutathione can be used as a marker for evaluating lipid peroxidation in heart failure^34^. Glutathione metabolism is also involved in endoplasmic reticulum stress and oxidative stress, and 5-oxoproline, the final product of Chac1 enzyme involved in metabolism, is also an important factor leading to heart failure^35^.In addition, in recent years, studies have found that ferroptosis is also associated with the occurrence of heart failure, which is related to the excessive production of ROS and the inhibition of glutathione synthesis by GPX4^36^. The primary physiological importance of cytochrome P450 enzymes lies in their ability to metabolize arachidonic acid to epoxyeicosatrienoic acid and hydroxyeicosatetraenoic acid metabolites, which play a role in maintaining cardiac homeostasis^37,38^. In short, in the KEGG enrichment results, DEGs mainly interfere with heart failure by affecting metabolism-related pathways.

Among the GSEA enrichment results, extracellular matrix organization and Diseases of metabolism were mainly involved in heart failure. The cardiac extracellular matrix contains a dynamic molecular network that provides structural support to cardiac tissue. Its renewal is an important process of cardiac remodeling. Excessive activation of pro-inflammatory cytokines such as TNFα promotes this process^39^. Therefore, the extracellular matrix can not only be used as an indicator to evaluate cardiac homeostasis, but also can be used to design more effective anti-fibrotic cardiac regeneration strategies^40^. From the results of enrichment analysis, the molecular mechanism of heart failure is closely related to inflammation, metabolism, oxidative stress and immunity.

Immune cell infiltration analysis showed that the degree of infiltration of Tgd cells and Neurons was significantly enriched in heart failure samples, whereas pDCs and NKTs were in healthy tissue samples. Cytotoxicity is the main biological effect of Tgd cells. Studies have shown that IL-17A secreted by Tgd cells can induce cardiomyocyte apoptosis^41^. By regulating the IL-17A/Tgd cells axis, it can effectively regulate the level of inflammation and slow down the process of myocardial fibrosis^42,43^. The enrichment of Neurons in heart failure samples may be related to the increase of cardiac sympathetic nerve activity and the enhancement of N-type Ca^2 + ^currents^44^. pDC and NKT are higher in healthy sample tissues, which is slightly different from previous studies^45–47^, and the specific mechanism behind it remains to be further studied.

According to PPI network analysis, we obtained Hub genes, including EGR1, EGR2, FOS and FOSB. EGR1 is widely present in human cells and has the ability to express rapidly, playing an important role in cell growth, differentiation, proliferation, and inflammatory responses^48^. Studies have shown that inhibiting the expression of EGR1 can slow myocardial fibrosis and thus treat heart failure^49^. EGR2, which belongs to the EGR family, has pro-inflammatory and pro-apoptotic effects. EGR2 can directly activate the expression of pro-apoptotic proteins such as BCL2 family and BINP3L, and can also upregulate inflammatory proteins such as IFNG and IL6^50^. Studies have shown that inhibition of EGR2 expression via lncRNA and miRNA can alleviate cardiac apoptosis and protect the heart from ischemic injury^51,52^. FOS and FOSB belong to the FOS gene family and are regulators of cell proliferation, differentiation, and transformation. In the early stage of cardiac remodeling, c-fos and c-jun were up-regulated, and the two dimerized to form the transcription factor complex AP-1, followed by the up-regulation of inflammatory factors NF-kappa B and NFAT. In late stage, genes involved in cardiac hypertrophic response such as ANP and BNP are up-regulated^53,54^. Therefore, the FOS family can be used as an early diagnostic marker of cardiac remodeling and a target for early intervention in the treatment of heart failure.

The novelties of our study are as follows. First, we analyzed the molecular mechanisms of heart failure related to immunity, inflammation, and metabolism through bioinformatics. Second, we identified EGR1, EGR2, FOS and FOSB as potential diagnostic biomarkers and therapeutic targets for heart failure. The diagnostic model based on these 4 genes provides a new idea for our future research on the molecular mechanism of heart failure. Nevertheless, this study still has some shortcomings. First, we were unable to determine whether there was a causal relationship between differences in gene expression and the physiopathological mechanisms of heart failure. Secondly, myocardial tissue sampling is often ranked after peripheral blood examination and imaging examination in clinical practice, and the sampling cost is high and the clinical value is limited. Finally, the lack of medication and prognostic data in the GEO database limited further analysis of differential genes. Although our results were validated in two datasets, further clinical trials are needed to confirm our findings.

## Conclusion

In summary, we explored the underlying molecular mechanisms of heart failure from the enrichment analysis and immune cell infiltration environment by bioinformatics analysis of the GEO dataset, which provided new ideas for the pathogenesis and treatment of heart failure. Through PPI network and logistic regression, we identified EGR1, EGR2, FOS and FOSB as potential diagnostic biomarkers and therapeutic targets for heart failure. More importantly, a diagnostic model of heart failure based on these 4 genes was developed, which leads to a new understanding of the pathogenesis of heart failure and may be an interesting target for future in-depth research.

## Data Availability

Publicly available datasets were analyzed in this study. This data can be found here: GSE8331; GSE76701; GSE21610; GSE57338.
